# Estimation risk of lymph nodal invasion in patients with early-stage cervical cancer: Cervical cancer application

**DOI:** 10.3389/fonc.2022.935628

**Published:** 2022-08-12

**Authors:** Benedetta Guani, Thomas Gaillard, Ly-Ann Teo-Fortin, Vincent Balaya, Anis Feki, Xavier Paoletti, Patrice Mathevet, Marie Plante, Fabrice Lecuru

**Affiliations:** ^1^ Department of Gynecology, Centre Hospitalier Universitaire Vaudois (CHUV), Lausanne, Switzerland; ^2^ Department of Gynecology, Hopital Fribourgeois (HFR), Fribourg, Switzerland; ^3^ Faculty of Biology and Medicine, University of Fribourg, Fribourg, Switzerland; ^4^ Department of Gynecology, Institut Curie, Paris, France; ^5^ Faculty of Medicine, Laval University of Quebec, Quebec, QC, Canada; ^6^ Department of Gynecology and Obstetrics, FOCH Hospital, Suresnes, France; ^7^ Department of Statistics, Institut Curie, Paris, France; ^8^ Faculty of Biology and Medicine, University of Lausanne, Lausanne, Switzerland; ^9^ Division of Gynecologic Oncology, Centre Hospitalier Universitaire (CHU) de Quebec, L’Hôtel-Dieu de Quebec, Quebec, QC, Canada; ^10^ Department of Medicine, University of Paris, Paris, France

**Keywords:** cervical cancer, lymph nodal status, early-stage cervical cancer, cervical cancer web application, gynecological cancer

## Abstract

**Introduction:**

Lymph node status is a major prognostic factor in early-stage cervical cancer. Predicting the risk of lymph node metastasis is essential for optimal therapeutic management. The aim of the study was to develop a web-based application to predict the risk of lymph node metastasis in patients with early-stage (IA1 with positive lymph vascular space invasion, IA2 and IB1) cervical cancer.

**Materials and methods:**

We performed a secondary analysis of data from two prospective multicenter trials, Senticol 1 and 2 pooled together in the training dataset. The histological risk factors were included in a multivariate logistic regression model in order to determine the most suitable prediction model. An internal validation of the chosen prediction model was then carried out by a cross validation of the ‘leave one out cross validation’ type. The prediction model was implemented in an interactive online application of the ‘Shinyapp’ type. Finally, an external validation was performed with a retrospective cohort from L’Hôtel-Dieu de Québec in Canada.

**Results:**

Three hundred twenty-one patients participating in Senticol 1 and 2 were included in our training analysis. Among these patients, 280 did not present lymph node invasion (87.2%), 13 presented isolated tumor cells (4%), 11 presented micrometastases (3.4%) and 17 macrometastases (5.3%). Tumor size, presence of lymph-vascular space invasion and stromal invasion were included in the prediction model. The Receiver Operating Characteristic (ROC) Curve from this model had an area under the curve (AUC) of 0.79 (95% CI [0.69– 0.90]). The AUC from the cross validation was 0.65. The external validation on the Canadian cohort confirmed a good discrimination of the model with an AUC of 0.83.

**Discussion:**

This is the first study of a prediction score for lymph node involvement in early-stage cervical cancer that includes internal and external validation. The web application is a simple, practical, and modern method of using this prediction score to assist in clinical management.

## Introduction

Lymph node status is a major prognostic factor for early-stage cervical cancer patients. The presence of lymph node metastasis was included in the latest revision of the International Federation of Gynecology and Obstetrics (FIGO) 2018 classification (1). The assessment of lymph node status is crucial for determining the most appropriate therapeutic strategy. Several publications have demonstrated the concept, feasibility, and validity of the Sentinel Lymph Node (SLN) technique in early-stage cervical cancer (2–7). According to the National Comprehensive Cancer Network (NCCN) guidelines (8), the SNL technique is considered as an alternative to pelvic dissection in early-stage cervical cancer. In the European Society of Gynecological Oncology (ESGO) guidelines (9), SLN biopsy (without additional pelvic lymph node dissection) is an acceptable method of lymph node staging only in stage IA. The major advantages of the SLN biopsy include a better quality of life (10) and reduced postoperative morbidity (11). Normally, the SLN was analyzed by ultrastaging pathological analysis in order to identify low-volume lymph node metastases: micrometastases (MIC) (0.2–2 mm) and isolated tumour cells (ITC) (<0.2 mm), which can be missed during conventional pathologic examination (12). In addition, low-volume lymph node metastases are rarely detected during frozen section analysis (13). In the new 2018 FIGO classification (1), patients with macrometastases (MAC) (>2 mm) or MIC are classified as stage IIIC1 in cases of pelvic involvement or IIIC2 in cases of lumbo-aortic involvement, whereas the presence of ITC does not change the stage of disease (1). The clinical impact and management of low-volume metastases are not fully understood and are still subject to debate (14), but results of a recent meta-analysis suggest that the presence of MICs is associated with a negative impact on disease-free survival and overall survival (15). Therefore, according to the 2018 FIGO classification (1) and the results of the above meta-analysis, MIC and MAC are considered as positive lymph nodes (N+). Our goal was to develop a web Application with a prediction score for lymph node involvement in early-stage cervical cancer. An internal and external validation was performed, in order to confirm the validity and reproducibility of our application.

## Materials and methods

### Population

We performed a secondary analysis of data from two prospective multicenter trials evaluating the place of the sentinel node in the surgical management of cervical cancer (Senticol 1 and 2) (2, 11). The inclusion criteria were as follows: patients older than 18 years with a diagnosis of early-stage cervical cancer, i.e. stage IA1 with vascular emboli to stage IIA according to the FIGO 2009 classification (16) with negative lymph nodes in the pre-operative scan. These patients were prospectively enrolled in seven French gynecologic oncology centers between 2005 and 2007 for the Senticol 1 study (2) and in 23 French centers between 2009 and 2012 for the Senticol 2 study (11). From these two prospective, multicenter databases, the individual clinical, radiological, and histological data necessary for our analyses were extracted. Tumor size and stromal invasion were obtained from the final pathologic analysis. The patients included in both studies had given their written consent to the use of the data for secondary analyses.

Finally, an external validation was performed using a retrospective cohort of patients treated at the CHU de Quebec, l’Hôtel-Dieu de Québec in Canada. Data were provided by CHU de Québec-Université Laval’s biobank. The included patients from the Canadian cohort were older than 18 years and had early-stage cervical cancer, i.e. stages IA1 with positive lymph vascular space invasion (LVSI), IA2 and IB1 according to the FIGO 2009 classification (16). All patients had given written consent to use their data. Sentinel node biopsies and lymphadenectomy surgeries had been performed on all patients. From the Canadian database, lymph node status, tumor size, stromal invasion and LVSI data were extracted for the App’s external validation. These data were then appropriately labelled and transferred to University Hospital of Vaud (CHUV, Lausanne, Switzerland) through a Redcap account.

### Surgical and pathological technique

In our cohort (Senticol 1 and 2), sentinel node detection was performed using a combined labelling technique: radioactive tracer (99mTc) and patent blue. Analysis of frozen sections was performed either systematically or only on nodes suspected of metastasis, at the discretion of the surgeon. Sentinel nodes of all patients were analyzed after staining with hematoxylin and eosin on 200-μM sections. All patients of Senticol 1 had SLN biopsy and following lymphadenectomy, all lymph nodes of the lymphadenectomy were secondarily analyzed by ultrastaging. In Senticol 2, one group of patients (group B) had SLN and lymphadenectomy and the second group (group A) had only SLN biopsy, without lymphadenectomy. In Senticol 2, only SLNs were subjected to ultrastaging, the other lymph nodes were analyzed with H&E.

In the Canadian cohort, all SLNs were analyzed by ultrastaging. The others lymph nodes were analyzed with H&E. Lymph node involvement was classified as follows: ITC for involvement less than 0.2 mm, MIC for involvement between 0.2 and 2 mm, and MAC for involvement greater than 2 mm. For node-negative patients, radical surgery was performed—either a radical hysterectomy or radical trachelectomy. Indications for adjuvant treatments were further determined following the final histology. For node-positive patients, radical surgery was abandoned in favor of definitive chemo-radiation following completion of laparoscopic para-aortic lymphadenectomy.

### Statistical analysis

Histological characteristics were compared for univariate analysis by chi2 tests or Fisher’s exact tests for qualitative data and by Student’s t-tests or ANOVA for quantitative data. The most clinically relevant variables, described by Sedlis (17), were entered into a multivariate logistic regression model to determine the best-fitting prediction model. The discriminatory ability of the model was represented with ROC curves and their associated area under the ROC curve (AUC) value. An internal validation of the selected prediction model was then performed by a ‘leave one out cross validation’. Finally, the prediction model was implemented in an interactive online application of the ‘Shinyapp’ type in open access. Shinyapp are web applications that allow access for all to applications derived from the RStudio software.

An external validation was then performed on an independent cohort. The external validation consisted in calculating the risk of lymph node involvement from the prediction model with the patients’ criteria. Then, we measured the concordance index (c-index) between the predicted risk of lymph node involvement and the pathologic node status. All statistical analyses were performed using RStudio version 4.0.1. The study obtained the approval of the Vaud Ethics Committee (CER-VD 2021-00780).

## Results

### Patients’ selection

**Figure 1:**
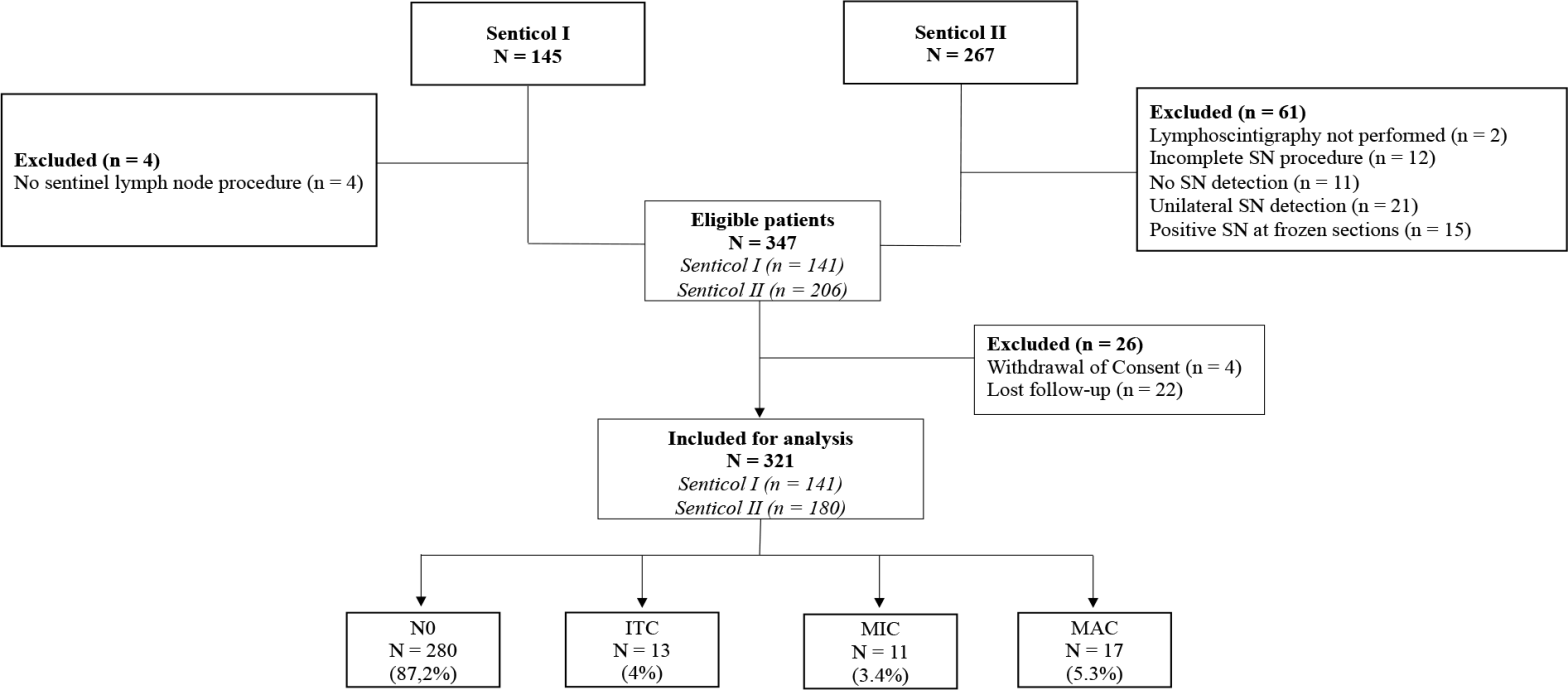
Flowchart of Senticol 1 and 2 patients inclusion. SN, sentinel node; N0, negative node; ITC, isolated tumour cells; MIC, micrometastasis; MAC, macrometastasis.

Among the patients included in the Senticol 1 and 2 studies, 321 patients were included in us analysis. Patients with no bilateral SLN detection, were excluded. The flowchart with detailed inclusions is available in the **Figure 1**. Of these patients, 280 had no lymph node invasion (N0 – 87.2%), 13 had ITC identified on immunohistochemical analysis (4%), 11 had lymph nodal MIC (3.4%) and 17 had lymph nodal MAC (5.3%).

### Senticol 1 and 2 patients risk factors

We divided the patients into two groups: N0 for the group without lymph node metastasis or with ITC (N0 and N0 [i+]) and N+ for the group with micro- and macro-metastatic lymph nodes (N+ and N+ [m]). Tumor characteristics according to the status of lymph node invasion is shown in **Table 1**. There was no statistically significant difference between the two groups in terms of histological type (p = 0.093), tumor grade (p = 0.25) and tumor size (p = 0.22). In the univariate analysis, the only statistical risk factors for lymph nodal invasion were the presence of LVSI (p = 0.0012) and stromal invasion (p = 0.03).

**Table 1:** Characteristics of Senticol 1 and 2 patients.

Risk factors		N0 = N0 and N0 (i+)		N+ = N+ and N+ (mi)	P stat
		(N = 293)		(N = 28)	
**Histological type**	Squamous	199 (67.9%)		24 (85.7%)	0.093
	Adenocarcinoma	83 (28.3%)		3 (10.7%)	
	Adeno-squamous	7 (2.4%)		0 (0%)	
	Other	4 (1.4%)		1 (3.6%)	
**Grade**	1	92 (31.4%)		7 (25%)	0.25
	2	76 (25.9%)		6 (21.4%)	
	3	33 (11.3%)		6 (21.4%)	
	*NA*	*92 (31.4%)*		*9 (32.1%)*	
**Tumour size**	<20 mm	180 (61.4%)		13 (46.4%)	0.22
	≥20 mm	91 (31.1%)		11 (39.3%)	
	*NA*	*22 (7.5%)*		*4 (14.3%)*	
	Median (mm)	15.04 (+/- 11.4)		18.41 (+/- 15.4)	0.26
**Stromal invasion**	}10 mm	112 (38%)		5 (17.8%)	0.03
	>10 mm	53 (18.1%)		8 (28.6%)	
	NA		128 (43.7%)	15 (53.6%)
	Median (mm)		9.4 (+/- 7.4)	13.5 (+/- 8)	0.063
**LVSI**	Negative		216 (73.7%)	13 (46.4%)	0.0012
	Positive		72 (24.6%)	15 (53.6%)	
	*NA*		*5 (1.7%)*	*0 (0%)*	

LVSI, lymph nodal space invasion; N0, negative lymph node; N0(i+), presence of isolated tumour cells in the lymph nodes; N+, positive lymph nodes; N+(mi), presence of micrometastasis in the lymph nodes; NA, not available.

We analyzed the relationship between the risk factors included in the Sedlis Criteria (17) and the presence of N+: only the positive LVSI seemed to be associated with the presence of lymph node metastasis (MAC and MIC) (OR = 16.9 [2.7–331.6], p = 0.01). There was no significant association between the depth of stromal invasion (in mm – OR = 0.32 [0.05–1.28], p = 0.93) or tumor size (in mm, OR = 1.01 [0.94–1.08], p = 0.74) and the presence of lymph node metastasis (**Table 2**).

**Table 2:** Odds Ratio analysis of risk factors included in Sedlis Criteria in Senticol 1 and 2 patients.

	Odds Ratio (OR [IC95%])		P stat
**LVSI**	Negative	1	
	Positive	16.9 [2.7–331.6]	0.01
**Stromal invasion (mm)**	/1 mm	0.32 [0.05–1.28]	0.93
**Tumour size (mm)**	/1 mm	1.01 [0.94–1.08]	0.74

LVSI, lymph nodal space invasion.

**Figure 2:**
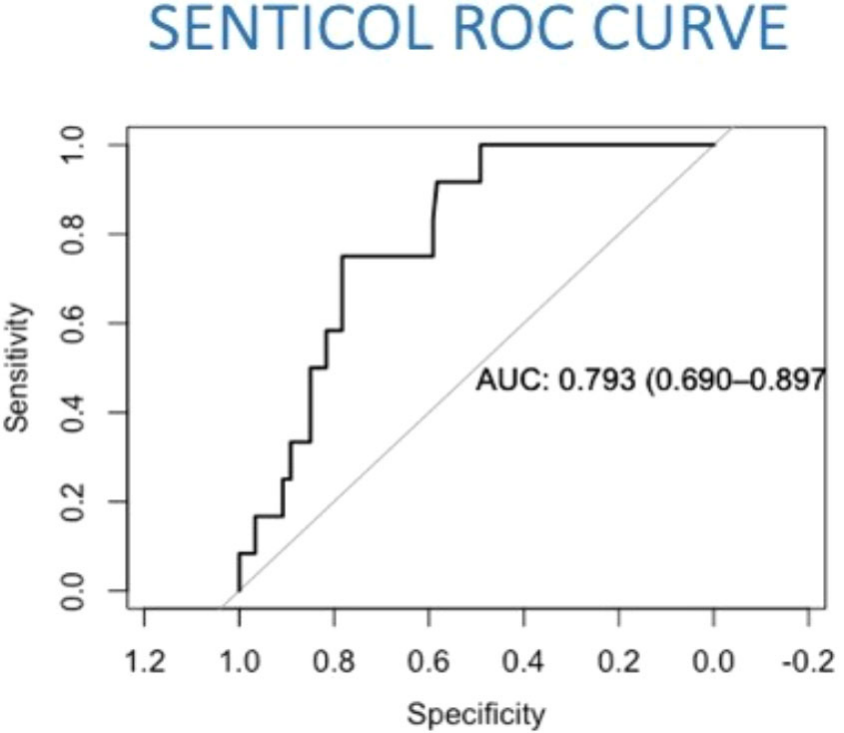
Prediction model of lymph nodal invasion in Senticol 1 and 2 patients. AUC, area under the curve; ROC, Receiver Operating Characteristic.

The predictive model of lymph nodal invasion in Senticol 1 and 2 patients included the 3 Sedlis Criteria showed an area under the curve (AUC) of 0.79 (95% CI [0.69–0.90]) (**Figure 2**).

### Internal validation

**Figure 3:**
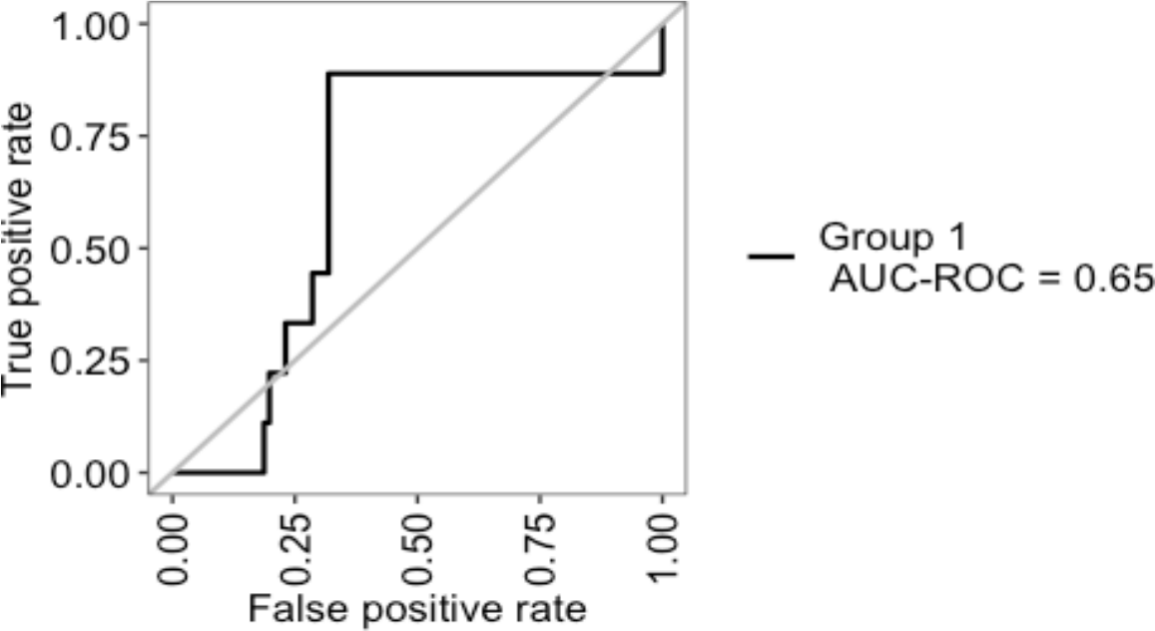
Internal validation of Senticol 1 and 2 patients by ‘leave-one-out cross validation’(LOOCV). AUC, area under the curve; ROC, Receiver Operating Characteristic.

An internal validation of the prediction model was then carried out by cross-validation using the so-called ‘leave-one-out cross validation’ (LOOCV) technique. Patients with missing values for one of the variables were excluded from cross-validation. The Receiver Operating Characteristic (ROC) curve resulting from the cross-validation is shown in **Figure 3**. The area under the curve (AUC) was 0.65.

### External validation

**Figure 4:**
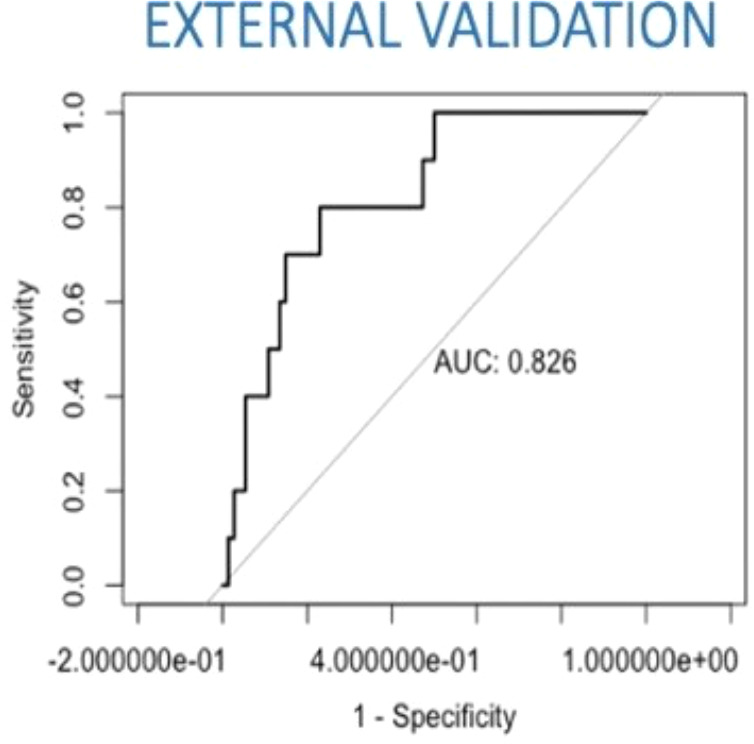
External validation with a Canadian population. AUC, area under the curve.

For the external validation, 100 patients were randomly selected amongst a list of patients treated for early-stage cervical cancer at Hôtel-Dieu de Québec, between 2008 and 2017. Furthermore, 15 out of the 100 selected patients (15%) had MAC, MIC, or ITC. The AUC of the external validation and the concordance index was 0.8256757 (**Figure 4**).

### CER-CAP web-application

**Figure 5:**
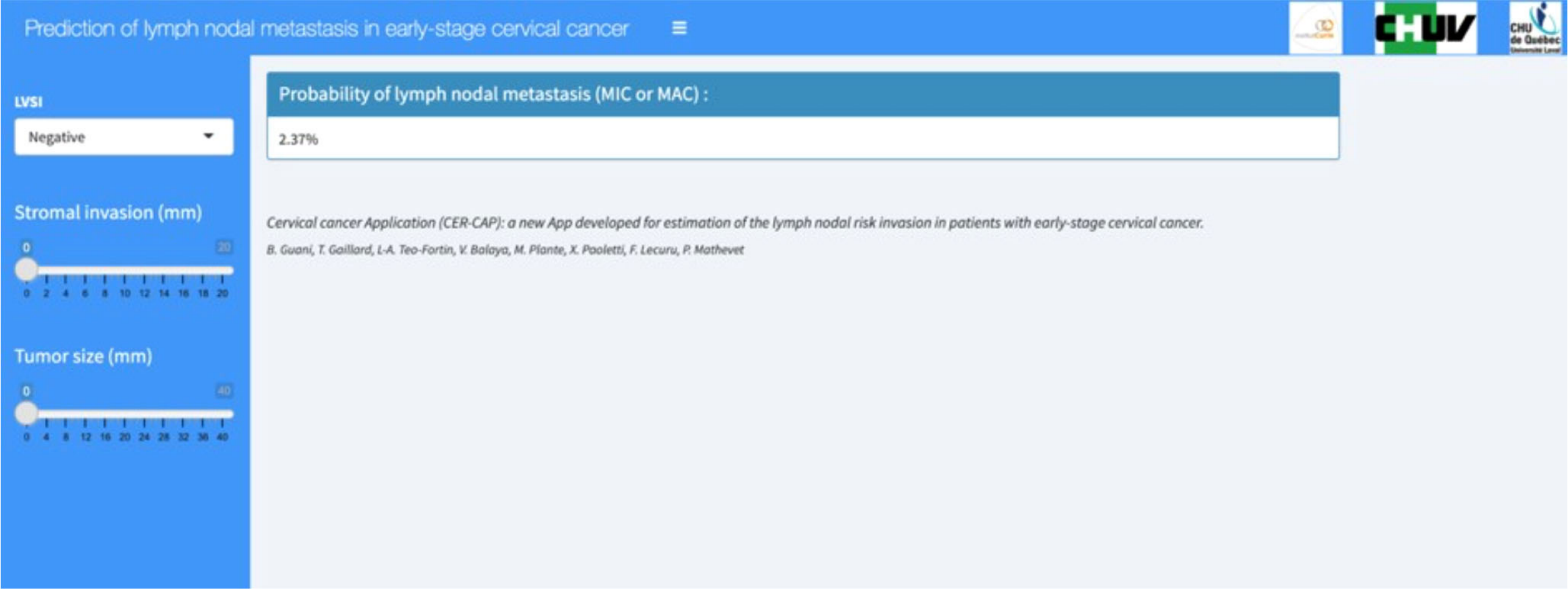
CER-CAP webpage screenshot. LVSI, lymph vascular space invasion; MIC, micrometastasis; MAC, macrometastasis.

Following this validation, a web application was created after extracting the logistic regression model. This web application makes it possible to predict an individual risk of lymph node metastasis from the coefficients from the model. The application thus calculates the probability of lymph node metastasis as a percentage (screenshot of the webpage in the **Figure 5**). This application is available online at the following address: https://thomas-gaillard.shinyapps.io/senticol_node_pred/?_ga=2.181936736.678230353.1655747040-1130361826.1585828032

### Groups of lymph nodal invasion risk

**Figure 6:**
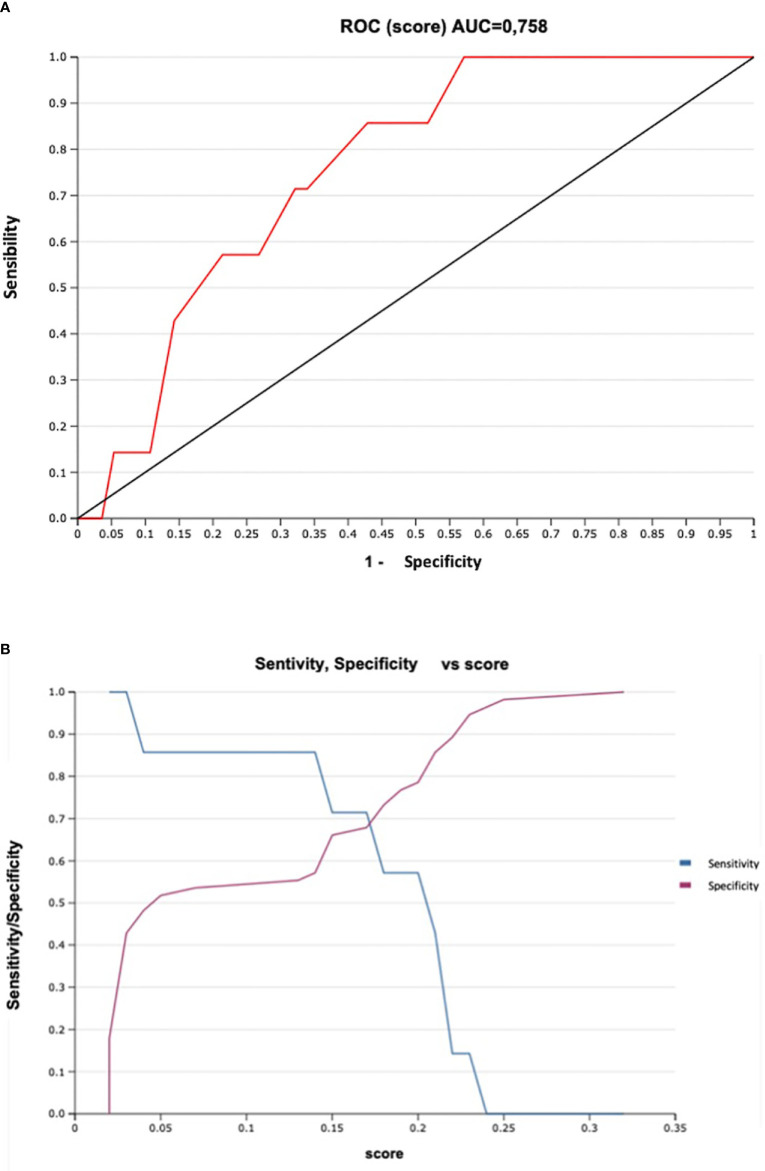
Sensitivity and Specificity of CER-CAP score: **(A)**. ROC Curve, ROC, Receiver Operating Characteristic; AUC, area under the curve. **(B)**. Sensitivity/Specificity vs score.

We were finally able to divide patients into risk groups of lymph node metastasis according to the score obtained by our predictive model. The CER-CAP score identified two groups: low-risk and high-risk (**Table 3**). According to the Senticol 1 and 2 population, a CER-CAP score indicating a low risk (<15%) identified 86% of N0 patients with none of the patients with MAC being in the low-risk category, while a CER-CAP score indicating a high risk identified 100% of patients with MAC. The CER-CAP score AUC was 0.76 (**Figure 6A**), the threshold value of >0.15 gives a good performance index to the test with a sensitivity of 85% and a negative predictive value of 97% (**Figure 6B**).

**Table 3:** Groups of lymph nodal invasion risk depending on the CER-CAP score, according to Senticol 1 and 2 patients.

CER- CAP score		N0	N0 (i+)	N+ (MAC)	N+ (mi)	Comment
***Low-risk***	<15%	95/110	4/7	0/9	2/5	86% of N0 (N0+ITC), 0 MAC
***High-risk***	>15%	15/110	3/7	9/9	3/5	85% of N+ (MAC+MIC),100% of MAC

N0, node negative; N0(i+), isolated tumor cells; N+, node positive; MAC, macrometastasis; N+(mi), micrometastasis

## Discussion

The primary objective of this study was to develop an online Application for clinical management of early-stage cervical cancer patients. For the predictive model, we used a sample of population (Senticol 1 and 2 population), then we performed an internal and an external validation.

### Summary of main results

A clinical score is based on a sample of the population, Senticol 1 and 2. In Senticol 1 and 2 the SLN mapping was performed with radioactive tracer (99mTc) and patent blue. Nowadays using indocyanine green seems a better choice with higher detection rates. In order to exclude this selection bias, only patients with bilateral detection of SLN were included. In Senticol 1 all lymph nodes were analyzed by ultrastaging, SLNs and non SLNs. In Senticol 2 and in the Canadian cohort, only the SLNs had ultrastaging analysis. We consider that these groups of patients are comparable. In fact, in the case of optimal mapping with bilateral detection, the NPV of SLN biopsy is 100% (18). We decided to include in our model variables known in the literature to be associated with lymph node involvement, despite the lack of significance in our study. Indeed, it seems increasingly recommended to include variables of interest known in the literature in multivariate models (19). Internal validation is indicated to determine its qualities when applied to the same sample. Cross-validation is a resampling method for estimating the reliability of a model. It uses different portions of the data to test and train a model on different interactions. Leave one out cross-validation (LOOCV) is a special case of cross-validation. This method is particularly suitable when the event being measured is rare.

In our case, the constructed prediction model performed very well (AUC 0.85), but the internal validation showed an AUC of 0.65. The explanation for the lower AUC is the presence of missing data between the variables in the model. In fact, only one-third of the original cohort (Senticol 1 and 2) showed all requested variables with no missing data. For this reason, our results required external validation with a large cohort, in order to accurately measure the relevance of our model. External validity consists in determining the qualities of the model when it is applied to another sample of the same population (reproducibility of the clinal score) or a different population (transportability of the clinical score) (20). In our study, the external validation included a sample of Canadian population, different from the European population, with a higher prevalence of lymph node metastasis. We measured the concordance index between the predicted risk and the pathologic node status.

The results were concordant with a very good discrimination of the model (AUC/C-index of 0.83). These results confirm that our predictive model is also transportable to other populations.

### Results in the context of published literature

To our knowledge, in the literature, there are no other validated scores to date for analyzing the risk of lymph node invasion in cervical cancer. On the other hand, there are many studies on the Kanagawa Cancer Center (KCC) preoperative scoring system (21, 22) and other systems (23) proposed for prediction of lymph nodes invasion in the endometrial cancer. The KCC preoperative scoring system is useful to predict lymph nodal metastases risk, and thereby prevent unnecessary lymphadenectomy or to determine its extent in endometrial cancer patients.

Our score includes the low-volume metastases. The low-volume metastases are unlikely to be detected by preoperative imaging such as magnetic resonance imaging (MRI) and are usually detected by ultrastaging of the sentinel node. The most important limitation of the ultra-staging technique is that it cannot be performed intraoperatively as it is too cumbersome and time-consuming. In addition, the frozen section (FS) accuracy for the detection of MIC is low: in Senticol 1 and 2 cohorts, the sensitivity and the negative predictive value of FS was 42.3% and 89.7%, respectively, for all types of SLN metastases or 56.4% and 94.1% if ITCs were excluded (13). The one-step nucleic acid amplification (OSNA) assay has recently been investigated in several tumour types, including cervical cancer patients (24), but for the moment the technique is not yet validated and remains to be confirmed by clinical studies. To date, the safest or ideal therapeutic strategy would consist of a two-step intervention with SLN ultrastaging analysis as a first step and then the radical (or conservative) surgery as a second step if SLN is negative (25, 26). This strategy must be counter-balanced by the increasing costs and processing times in case of N0, and it requires two hospitalizations and surgeries for patients. In addition, the second surgery (usually 10 days later), can be more difficult because of inflammation and postoperative adhesions. With our CER-CAP Application, we would like to provide a practical tool for programming the surgical management of patients with early-stage cervical cancer. Indeed, in case of high-risk of nodal invasion according to the CER-CAP score, we propose to perform the lymph node evaluation first and wait for the definitive results of ultrastaging before deciding on management. In case of a low-risk score, we propose to proceed directly to surgical treatment and avoid the morbidity of a two- step procedure. The false-negative rate of the CER-CAP score applied to the Senticol 1 and 2 cohort is low and allows detection >85% of N0 and N+, including MIC. If we analyze only the MAC (excluding MIC), the prediction of the score applied to Senticol 1 and 2 is 100% with no MAC detected in low-risk patients. In case of false negative of the CER-CAP score with discovery of a MIC in the SLN after the definitive pathological analysis, in a patient previously classified in low-risk, an adjuvant treatment of chemo-radiotherapy seems indicated.

In fact, as discussed in the meta-analysis (15), the adequate treatment of MIC (lymphadenectomy vs chemo-radiotherapy) remains unclear, due to the lack of evidence, a consequence of the low number of recurrences in the low-volume metastasis situation and in early-stage cervical cancer. However, if we compare cervical with vulvar cancer, the results of the recent Groningen International Study on Sentinel nodes in Vulvar cancer (GROINSS-V2) (27) showed that adjuvant radiotherapy treatment is a safe alternative in patients with vulvar cancer and lymph nodal MIC. Specific studies on cervical cancer are necessary in order to define the adequate treatment of MIC in cervical cancer.

### Strengths and weaknesses

CER-CAP is the first study of a prediction score for lymph node involvement in early-stage cervical Cancer. This online Application is a simple, practical and modern method of using this prediction score in clinical management, and it can be used to decide the clinical management of patients with early-stage cervical cancer. The Application can reduce the use of the Frozen Section which is often inaccurate and does not find micrometastases in a large number of cases.

To avoid problems related to FIGO stage, which is constantly evolving, the application requires to include tumor size, LVSI, and the numerical value of stromal invasion and not FIGO stage. Therefore, the App is applicable whatever FIGO classification is used and will also be applicable in the future, should it change.

The limitation of this study is the choice of the CER-CAP score (threshold value of 15%) which is based on the results of Senticol 1 and 2 studies, despite the presence of missing data between the variables in the model. However, the excellent results of the external validation confirm that us predictive model is transportable to other populations.

### Implications for practice and future research

We believe that the CER-CAP score can become a practical tool in the discussion of management during tumour boards and multidisciplinary meetings, in order to aid in clinical management.

### Conclusions

CER-CAP is the first study of a prediction score for lymph node involvement in early-stage cervical cancer that includes internal and external validation. The online Application can become a practical tool for clinical management of early-stage cervical cancer patients.

## Data availability statement

The raw data supporting the conclusions of this article will be made available by the authors, without undue reservation.

## Ethics statement

The studies involving human participants were reviewed and approved by Comité Ethique Canton Vaud, Lausanne, Switzerland. The patients/participants provided their written informed consent to participate in this study.

## Author contributions

Conceptualization, BG, FL, VB, and PM. Methodology, BG, TG, XP, and L-AT-F. Software, BG, TG, and XP. Validation, VB, FL, XP, MP, AF, and PM. Investigation, BG and L-AT-F. Resources, MP, PM, and FL. Data curation, BG and L-AT-F. Writing—original draft preparation, BG, TG, and L-AT-F. Writing—review and editing, VB, FL, MP, AF, and PM. Supervision, FL, MP, AF, and PM. All authors have read and agreed to the published version of the manuscript. All authors contributed to the article and approved the submitted version.

## Funding

Open access funding was provided by the University of Lausanne.

## Acknowledgments

We acknowledge all centers participating in the Senticol 1 and 2 studies and the Senticol Group Members. Completed list is in **Supplementary Material**.

## Conflict of interest

The authors declare that the research was conducted in the absence of any commercial or financial relationships that could be construed as a potential conflict of interest.

## Publisher’s note

All claims expressed in this article are solely those of the authors and do not necessarily represent those of their affiliated organizations, or those of the publisher, the editors and the reviewers. Any product that may be evaluated in this article, or claim that may be made by its manufacturer, is not guaranteed or endorsed by the publisher.
