# Erratum: Estimation risk of lymph nodal invasion in patients with early-stage cervical cancer: Cervical cancer application

**DOI:** 10.3389/fonc.2023.1178841

**Published:** 2023-03-24

**Authors:** Frontiers Production Office

**Affiliations:** Frontiers Media SA, Lausanne, Switzerland

**Keywords:** cervical cancer, lymph nodal status, early-stage cervical cancer, cervical cancer web application, gynecological cancer

## Introduction

An omission to the funding section of the original article was made in error. The following sentence has been added: “Open access funding was provided by the University of Lausanne”.

The original version of this article has been updated.

