# Supplementary material for: Estimation risk of lymph nodal invasion in patients with early-stage cervical cancer: Cervical cancer application
Source: Front Oncol. 2022 Aug 12;12:935628. doi: 10.3389/fonc.2022.935628 (PMC9413841; doi:10.3389/fonc.2022.935628)
Supplement: Supplementary file 1 [file DataSheet_1.docx]

Supplementary Material

# Supplementary Data: SENTICOL GROUP

# List of surgeons and centers participating in Senticol 1 and 2 trials

P. Mathevet, HFME Lyon, France

P. Morice, Institut Gustave Roussy, Villejuif, France

D. Querleu, Institut Claudius Régaud, Toulouse, France

E. Stoeckle, Institut Bergonié, Bordeaux, France

F. Lecuru, HEGP Paris, France

V. Fourchotte, Institut Curie, Paris, France

E. Leblanc, Centre Oscar Lambret, Lille, France

E. Daraï, Hôpital de Tenon, Paris, France

M. Baron, Centre Henri Becquerel, Rouen, France

H. Marret, Hôpital Bretonneau, Tours, France

J. Lévèque, Hôpital Sud Anne de Bretagne, Rennes, France

B. Ott, Hôpital du Hasenrain, Mulhouse, France

D. Lanvin, Clinique de l’Espérance, Mougins, France

C. Pomel, Centre Jean Perrin, Clermont Ferrand, France

G. Houvenaeghel, Institut Paoli-Calmettes, Marseille, France

P. Rouanet, Centre Val d’Aurelle, Montpellier, France

P. Descamps, Centre Hospitalier Universitaire, Angers, France

G. Mage, Centre Hospitalier Régional Universitaire, Clermont Ferrand, France

O. Graesslin, Institut Mère Enfant, Reims, France

JJ. Baldauf, Hôpital de Haute Pierre, Strasbourg, France

JM. Classe, Centre René Gauducheau, Nantes, France

D. Raudrant, Centre Hospitalier Lyon Sud, Lyon, France

V. Conri, Hôpital Pellegrin, Bordeaux, France

S. Douvier, Centre Hospitalier Universitaire, Dijon, France

Y. Delpech, Hôpital Lariboisière, Paris, France

P. Leguévaque, Hôpital Rangueil, Toulouse, France

Y. Fouché, Centre Antoine Lacassagne, Nice, France

L. Boulanger, Hôpital Jeanne de Flandre, Lille, France

A.S. Bats, Hôpital Européen Georges Pompidou, Paris, France

C. Uzan, Institut Gustave Roussy, Villejuif, France

F. Bouttitie, Service d’informatique, CHLS, Lyon, France

L. Magaud, HFME Lyon, France

B. Guani, CHUV Lausanne, Switzerland

V. Balaya, HEGP Paris, France
